# Polynucleotide Phosphorylase Mediates a New Mechanism of Persister Formation in Escherichia coli

**DOI:** 10.1128/spectrum.01546-22

**Published:** 2022-12-08

**Authors:** Nan Wu, Yumeng Zhang, Shanshan Zhang, Youhua Yuan, Shuang Liu, Tao Xu, Peng Cui, Wenhong Zhang, Ying Zhang

**Affiliations:** a Department of Clinical Laboratory, Shanghai Stomatological Hospital, Shanghai, China; b Department of Infectious Diseases, Shanghai Key Laboratory of Infectious Diseases and Biosafety Emergency Response, National Medical Center for Infectious Diseases, Huashan Hospital, Fudan University, Shanghai, China; c State Key Laboratory for the Diagnosis and Treatment of Infectious Diseases, The First Affiliated Hospital, Zhejiang University School of Medicine, Hangzhou, China; University of Arizona

**Keywords:** PNPase, *Escherichia coli*, persister mechanisms, cyclic AMP receptor protein, persister cells, knockout, metabolism, RNA degradation

## Abstract

Despite the identification of many genes and pathways involved in the persistence phenomenon in bacteria, the mechanisms of persistence are not well understood. Here, using Escherichia coli, we identified polynucleotide phosphorylase (PNPase) as a key regulator of persister formation. We constructed the *pnp* knockout strain (*Δpnp*) and its complemented strain and exposed them to antibiotics and stress conditions. The results showed that, compared with the wild-type strain W3110, the *Δpnp* strain had significant defects in persistence to antibiotics and stresses, and the persistence phenotype was restored upon complementation with the *pnp* gene. Transcriptome sequencing (RNA-seq) analysis revealed that 242 (166 upregulated and 76 downregulated) genes were differentially expressed in the *Δpnp* strain compared with the W3110 strain. KEGG analysis of the upregulated genes showed that these genes were mostly mapped to metabolism and virulence pathways, of which most are positively regulated by the global regulator cyclic AMP receptor protein (CRP). Correspondingly, the transcription level of the *crp* gene in the *Δpnp* strain increased 3.22-fold in the early stationary phase. We further explored the indicators of cellular metabolism of the *Δpnp* strain, the phenotype of the *pnp* and *crp* double-deletion mutant, and the transcriptional activity of the *crp* gene. Our results indicate that PNPase controls cellular metabolism by negatively regulating the *crp* operon via targeting the 5′-untranslated region of the *crp* transcript. This study reveals a persister mechanism and provides novel targets for the development of drugs against persisters for more effective treatment.

**IMPORTANCE** Persisters pose significant challenges for a more effective treatment of persistent infections. An improved understanding of mechanisms of persistence will provide therapeutic targets important for the development of better treatments. Since recent studies with the key tuberculosis persister drug pyrazinamide have implicated polynucleotide phosphorylase (PNPase) as a drug target, in this study, we addressed the possibility that PNPase might be involved in persistence in Escherichia coli. Our study demonstrates PNPase indeed being involved in persistence, provides a mechanism by which PNPase controls persister formation, and suggests a new therapeutic target for treating persistent bacterial infections.

## INTRODUCTION

Persisters are a small fraction of dormant or nongrowing bacteria that are tolerant to lethal antibiotics (1–3). Persisters are distinct from antibiotic-resistant cells in that they are genetically identical and remain susceptible to antibiotics when they resume growth (1, 4). Persisters pose significant challenges to the treatment of many chronic and persistent bacterial infections, such as tuberculosis, urinary tract infections, and biofilm infections (5–7). Therefore, it is of great importance to understand the mechanisms of persistence and to develop new strategies to more effectively cure such persistent infections (8, 9). Although the phenomenon of bacterial persistence was discovered over 70 years ago (10), our understanding of the molecular mechanisms of persister formation remains incomplete.

In our previous study of the peculiar tuberculosis persister drug pyrazinamide (PZA) (11), the active component of PZA, namely, pyrazinoic acid (POA), was found to bind to Mycobacterium tuberculosis PNPase, a bifunctional enzyme, guanosine pentaphosphate synthetase (GpsI)/polyribonucleotide nucleotidyltransferase (Rv2783) involved in RNA degradation (12), which was subsequently shown to be a new target of PZA (13, 14). Since PZA is a well-known persister drug (11), we wondered if PNPase as a target of persister drug PZA might be involved in persister formation and survival. In Escherichia coli, polynucleotide phosphorylase (PNPase) encoded by *pnp* is a 3′ to 5′ exonuclease and a 3′-terminal oligonucleotide polymerase and a major component of the RNA degradosome, which is composed of a complex structure with RNase E, helicase RhlB, and enolase together with PNPase (15, 16). In bacteria, mRNA degradation is of great significance, as it not only achieves nucleotide recycling but also can control gene expression under different growth conditions (17, 18). PNPase has been found to be important in many aspects of RNA metabolism (19–21). In addition, it also plays important roles in posttranscriptional regulation of gene expression (22).

In this study, to test the possibility that PNPase might be involved in persistence and to address the role of RNA degradation in bacterial persistence, we used E. coli as a model and constructed the *pnp* knockout mutant strain (*Δpnp*) and its complementation strain, and we assessed their survival upon exposure to antibiotics and stress conditions. In addition, transcriptome sequencing (RNA-seq) was performed to evaluate the transcriptome of the *Δpnp* strain compared with that of the parent strain W3110, and differential gene expression was analyzed to shed light on the molecular basis of the *pnp*-mediated persistence. We demonstrate that PNPase controls cellular metabolism by negatively regulating the global regulator cyclic AMP receptor protein (CRP operon at the posttranscriptional level through targeting the 5′-untranslated region [UTR] of the *crp* transcript) to regulate persister formation.

## RESULTS

### The *Δpnp* strain has a defect in persistence to various antibiotics.

**FIG 1:**
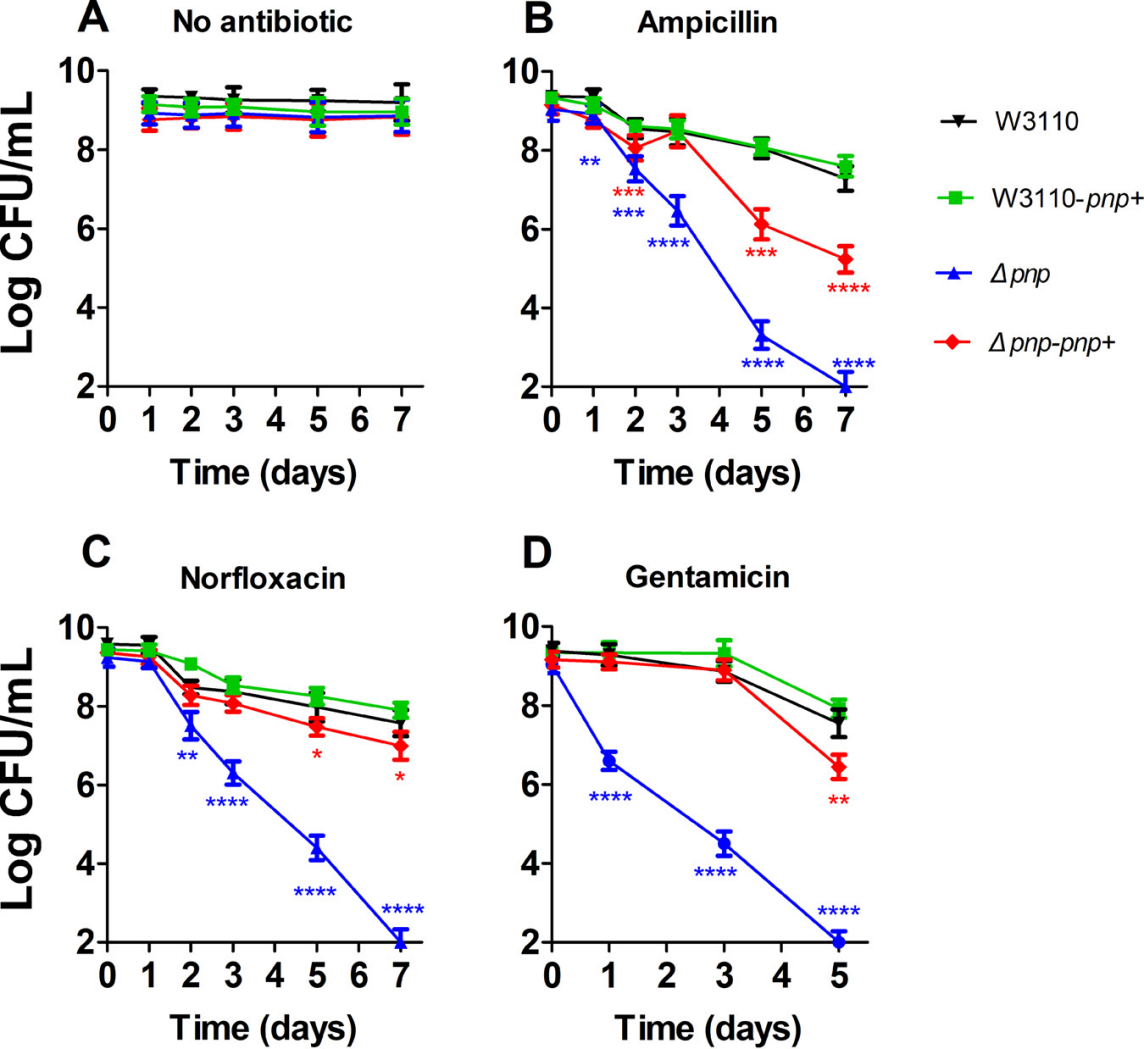
Defective persister levels of the *Δpnp* strain. Stationary-phase cultures of the *Δpnp* strain, its complemented strain (*Δpnp*-*pnp*+), the wild-type W3110 strain, and the *pnp* overexpression strain (W3110-*pnp*+) were exposed to no antibiotics (A), ampicillin (200 μg/mL) (B), norfloxacin (8 μg/mL) (C), and gentamicin (40 μg/mL) (D) for various times. Aliquots of cultures were taken at different time points, washed, and plated for CFU determination on LB plates. The vertical axis represents CFU values on a log scale, and the horizontal axis represents time of antibiotic exposure in days. Data are the average results from at least three independent experiments. Error bars represent standard deviation (SD). *, *P* < 0.05; **, *P < *0.01; ***, *P < *0.001; ****, *P < *0.0001 (Student’s *t* tests).

A *pnp* knockout strain (*Δpnp*) was constructed via homologous recombination. The growth rate of *Δpnp* was slightly slower than that of the wild-type strain W3110 in the logarithmic growth period. During the stationary phase, the number of bacteria in both groups reached the same order of magnitude (about 10^9^ CFU/mL) (see Fig. S1A in the supplemental material). In the motility assay, the *Δpnp* strain demonstrated a defect in movement in Luria-Bertani (LB) broth containing 0.1% agar, compared with W3110 and *Δdam* (a mutant involved in persistence but normal in the motility assay) as controls (Fig. S1B). In addition, the *Δpnp* strain had more biofilm formation when stained with crystal violet dye than the complemented or overexpression *pnp* mutant strains or W3110 (Fig. S1C). The stationary-phase cultures of the *pnp* mutant and the W3110 as a control were exposed to various antibiotics, including ampicillin (200 μg/mL), norfloxacin (8 μg/mL), and gentamicin (40 μg/mL). The results showed that the *Δpnp* strain was more susceptible than the W3110 strain to all the three antibiotics. Especially upon treatment with gentamicin, the persister levels of the *Δpnp* strain decreased significantly after 24 h of exposure. The effect of ampicillin and norfloxacin on sterilization was similar. The *Δpnp* strain was killed by ampicillin and norfloxacin significantly from day 2 time point. Complementation of the *Δpnp* strain with the functional *pnp* gene (*Δpnp-pnp+*) conferred increased persistence to the three antibiotics (Fig. 1; see Fig. S2 in the supplemental material). There was no significant change in the persister level between the *pnp* gene overexpression strain (W3110*-pnp+*) and wild-type strain W3110 (Fig. 1 and Fig. S2).

### The *Δpnp* strain is more susceptible to various stresses.

**FIG 2:**
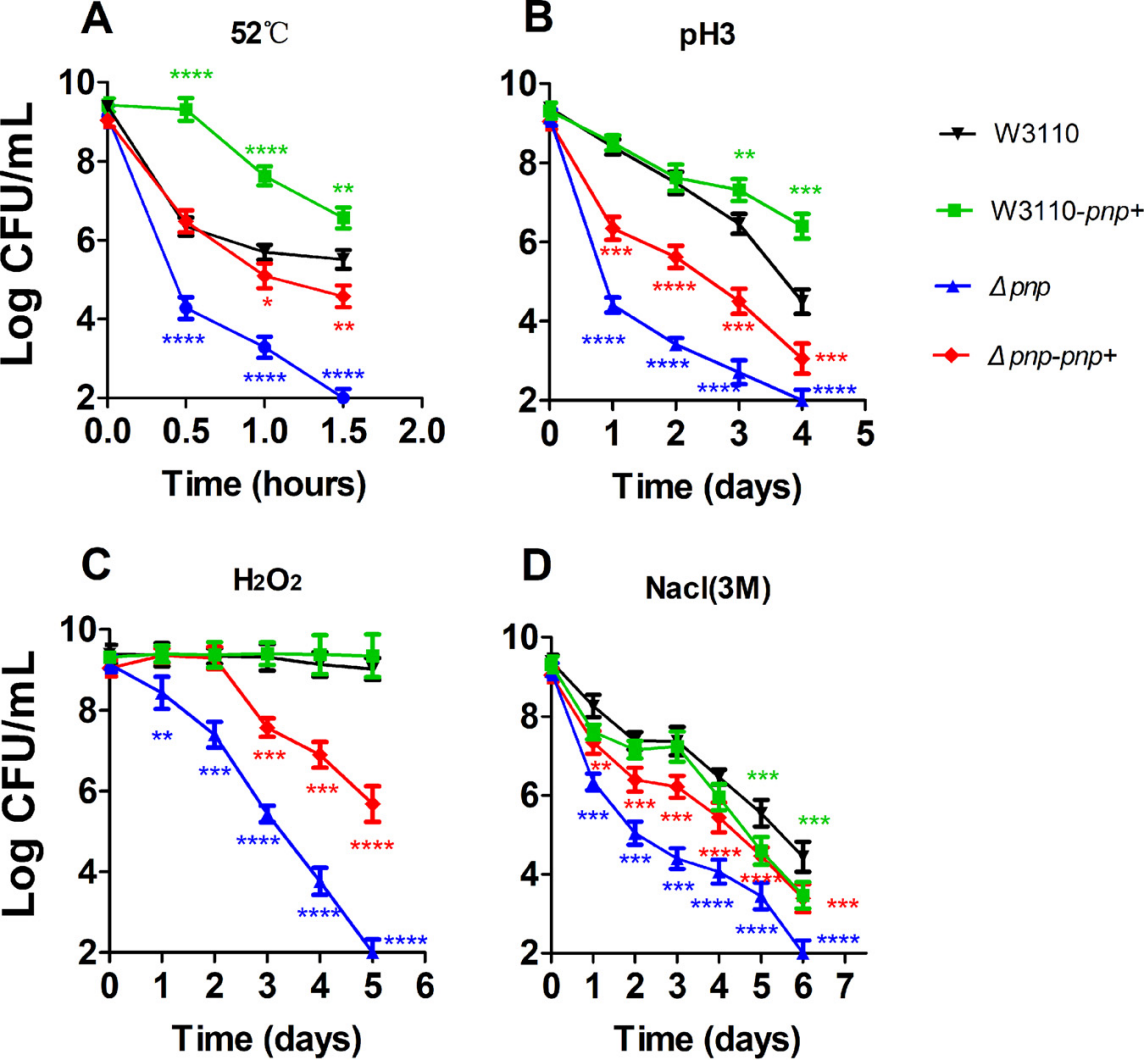
Increased susceptibility of the *Δpnp* strain to stresses. The *Δpnp* strain, its complemented strain (*Δpnp*-*pnp*+), the wild-type W3110 strain, and the *pnp* overexpression strain (W3110-*pnp*+) were exposed to different stress conditions, as follows: heat at 52°C (A), acid pH 3.0 (B), hydrogen peroxide (80 mM) (C), and high osmolarity (3 M NaCl) (D). All strains were cultured to stationary phase and exposed to different stresses followed by CFU determination. The vertical axis represents CFU values on a log scale, and the horizontal axis represents time of stress exposure in hours or days. Data are the average results from at least three independent experiments. Error bars represent standard deviation (SD). *, *P < *0.05; **, *P < *0.01; ***, *P < *0.001; ****, *P < *0.0001 (Student’s *t* tests).

Since the persister bacteria are tolerant not only to antibiotics but also to certain stress conditions (23), we also tested the survival of the *Δpnp* strain under several stress conditions, including heat, acid pH, hydrogen peroxide, and hypertonic saline stress. As shown in Fig. 2, the stationary-phase culture of the *Δpnp* strain was more sensitive to the four stress conditions than that of the W3110 (Fig. 2). Although the *Δpnp-pnp+* strain could not restore survival to wild-type levels, it is clear that *pnp* conferred a higher persistence level that allowed the complemented strain to better withstand treatments with different stresses. Moreover, it is worth noting that the persistence level was significantly higher in the *pnp* overexpression strains than that in the wild-type strains under heat stress (Fig. 2A) but was significantly lower in the *pnp* overexpression strains than that in the wild-type strains under hypertonic saline stress (Fig. 2D).

### RNA-seq analysis reveals a higher metabolic status of the *Δpnp* strain.

**FIG 3:**
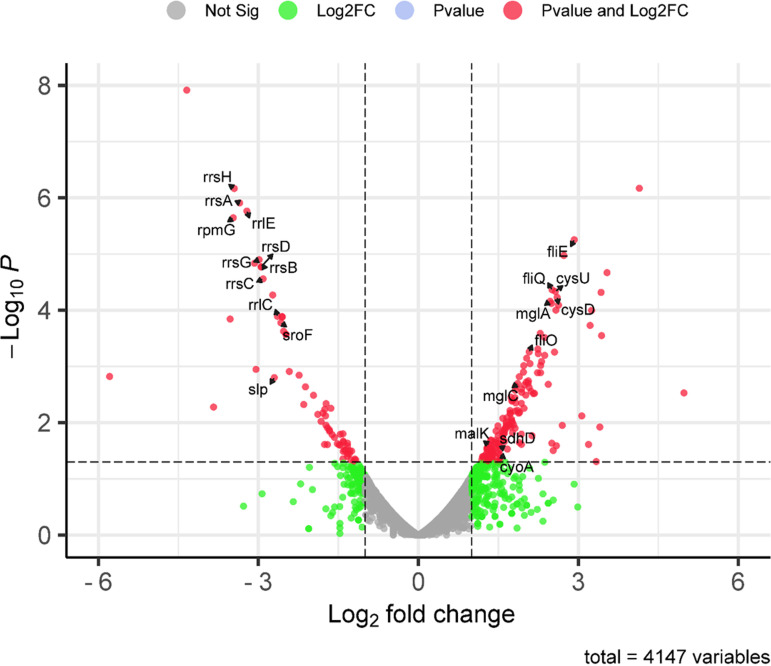
Volcano plot of differentially expressed genes (DEGs) between the *Δpnp* strain and the wild-type W3110 strain in early stationary phase. Volcano plot showing the distribution of significance [−log_10_(*P*-value)] versus fold change [log_2_(fold change)] for all genes. The upregulated genes selected for qPCR validation and downregulated genes enriched in ribosome pathway are highlighted and their gene symbols marked. Genes in red are significant DEGs [*P* ≤ 0.05 and log_2_(fold change) of ≥1].

The volcano plot (Fig. 3) shows the difference in gene expression profiling between the W3110 and the *Δpnp* strain. Altogether, 242 genes showed significant differences in the *Δpnp* strain compared with the W3110 strain, where 166 genes were upregulated and 76 genes were downregulated (see Table S1 in the supplemental material). In the volcano plot, 10 upregulated genes (*fliE*, *fliQ*, and *fliO* enriched in flagellar assembly; *malK*, *mglA*, and *mglC* enriched in ABC transporter; *cysD* and *cysU* enriched in sulfur metabolism; *cyoA* enriched in oxidative phosphorylation; and *sdhD* enriched in citrate cycle) selected for real-time quantitative PCR (qPCR) validation and 11 downregulated genes (*rpmG*, *rrsH*, *rrsA*, *rrlE*, *rrsG*, *rrsD*, *rrsB*, *rrsC*, *slp*, *rrlC*, and *sroF*) enriched in the ribosome pathway are highlighted with their gene symbols marked (Fig. 3).

**FIG 4:**
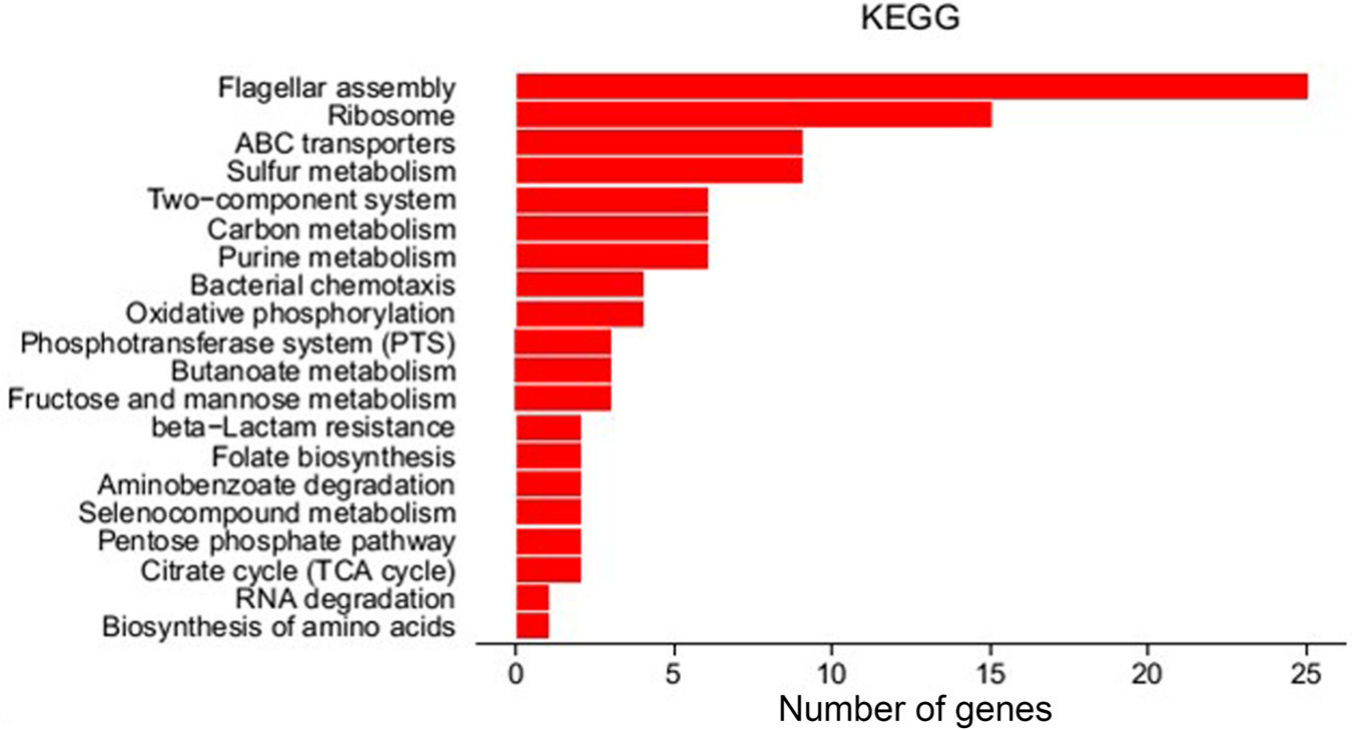
KEGG analysis of differential pathways between the *Δpnp* strain and the wild-type W3110 strain. Top 20 pathways are shown.

Pathway analysis of the differentially expressed genes (DEGs) was performed using the KEGG database, and the significance of the DEG enrichment in each pathway was calculated by the hypergeometric distribution test. The top 20 pathways after KEGG analysis are shown in Fig. 4. Interestingly, flagellar assembly (PATH ecj02040), ribosome (PATH ecj03010), ABC transporters (PATH 02010), sulfur metabolism (PATH ecj00920), two-component system (PATH ecj02020), and carbon (energy) metabolism (PATH ecj01200) are the main enrichment pathways of significant differences in gene transcripts, in which only the genes enriched in ribosome pathway are downregulated (see Table S2 in the supplemental material). We selected 41 genes whose mRNA levels increased more than 2-fold in the *Δpnp* strain for validation by qPCR. The results showed that the expression levels of 32 genes were upregulated more than 4-fold (Table 1). The expression trends of the other 9 genes showed no significant difference.

**TABLE 1:** Validation of differentially expressed genes (DEGs) in RNA-seq data between the *Δpnp* strain and the W3110 strain by qPCR

Gene	Fold change (*Δpnp*/WT)	Description	Pathway
*acnB*	7.71	Aconitate hydratase 2	Citrate cycle
*aldA*	4.01	Aldehyde dehydrogenase A	Pyruvate metabolism
*cyoA*	6.88	Cytochrome bo(3) ubiquinol oxidase subunit II	Oxidative phosphorylation
*cysD*	6.07	Sulfate adenylyltransferase, subunit 2	Sulfur metabolism
*cysU*	5.93	Sulfate/thiosulfate ABC transporter permease	Sulfur metabolism/ABC transporter
*fimC*	14.49	Periplasmic chaperone	
*fimD*	7.71	Fimbrial usher outer membrane porin protein	
*fimI*	9.06	Fimbrial protein involved in type 1 pilus biosynthesis	
*fliE*	7.58	Flagellar basal-body component	Flagellar assembly
*fliN*	4.72	Flagellar motor switching and energizing component	Bacterial chemotaxis/flagellar assembly
*fliO*	4.22	Flagellar biosynthesis protein	Flagellar assembly
*fliQ*	5.68	Flagellar biosynthesis protein	Flagellar assembly
*fumA*	4.71	Fumarate hydratase	Citrate cycle
*glpD*	5.15	Glycerol-3-phosphatedehydrogenase	Glycerophospholipid metabolism
*glpK*	4.59	Glycerol kinase	Glycerolipid metabolism
*glpT*	5.16	Glycerol-3-phosphate transporter	
*lamB*	5.36	Maltose outer membrane porin	
*malK*	4.25	Maltose ABC transporter ATPase	ABC transporter
*mdh*	4.68	Malate dehydrogenase	Cysteine and methionine metabolism/citrate cycle
*mglA*	5.54	ATP-binding cassette protein for galactose uptake	ABC transporter
*mglB*	6.77	Methyl-galactoside transporter subunit	Bacterial chemotaxis/ABC transporter
*mglC*	4.64	Galactose permease protein	ABC transporter
*nmpC*	4.58	Outer membrane porin protein	
*ompF*	9.32	Outer membrane protein F	Two-component system
*putP*	4.22	Sodium/proline symporter	
*sdhA*	6.53	Succinate dehydrogenase	Citrate cycle
*sdhD*	6.39	Succinate dehydrogenase, membrane subunit	Citrate cycle
*srlE*	4.08	Glucitol/sorbitol-specific enzyme IIB component of PTS	Phosphotransferase system
*srlD*	4.41	Sorbitol-6-phosphate dehydrogenase	Fructose and mannose metabolism
*xapA*	5.67	Purine nucleoside phosphorylase 2	Purine metabolism
*ydcA*	10.74	Uncharacterized protein	
*yfdF*	5.77	Uncharacterized protein	

### Detection of internal redox status of bacteria.

**FIG 5:**
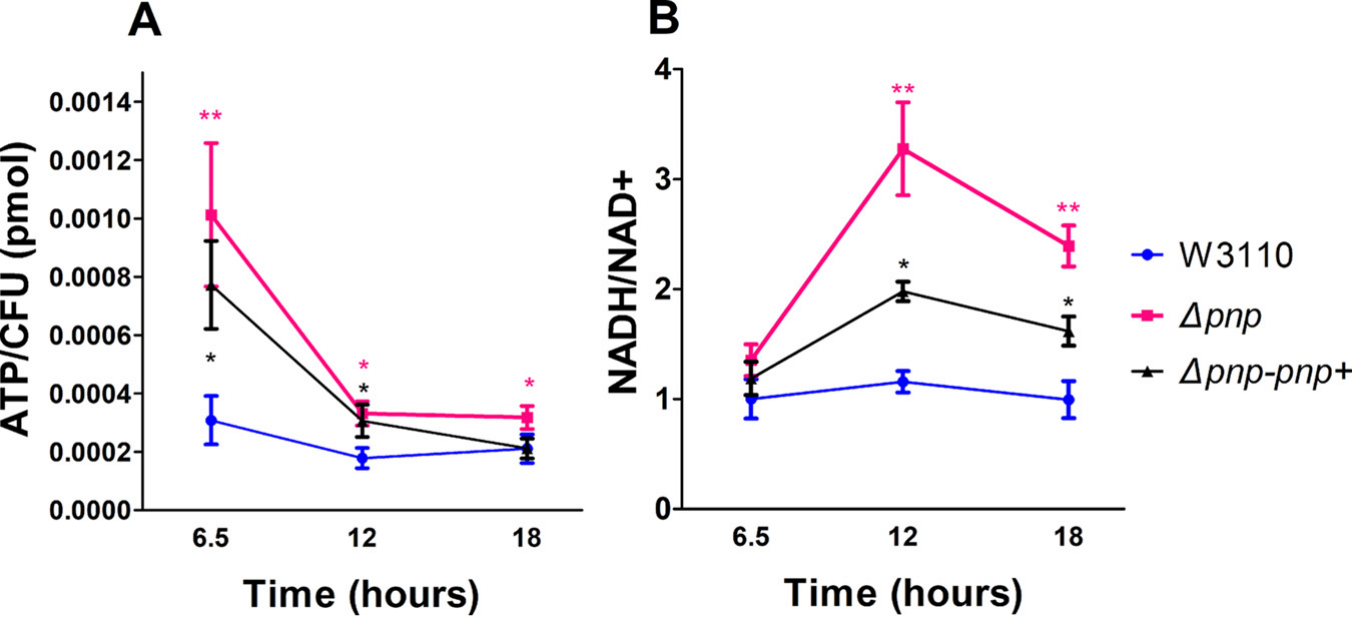
Detection of ATP and NADH/NAD^+^ in the *Δpnp* strain, its complemented strain (*Δpnp*-*pnp*+), and the wild-type W3110 strain. (A) The ATP levels at each stage of the W3110, the *Δpnp* strain, and the *Δpnp*-*pnp*+ strain were detected, and the absolute concentrations of ATP were calculated by the ATP standard curve. The time points 6.5 h, 12 h, and 18 h represent the early stationary phase, the middle stationary phase, and the end of stationary phase, respectively. (B) The NADH/NAD^+^ ratios of the W3110 strain, the *Δpnp* strain, and its complemented strain were measured. Data are the average results from at least three independent experiments. Error bars represent standard deviation (SD). *, *P < *0.05; **, *P < *0.01 (Student’s *t* tests).

Since the RNA-seq results showed that genes involved in energy metabolism and virulence-related pathways were expressed at a higher level in the *Δpnp* strain than those in the parent strain, we measured the intracellular ATP levels and NADH/NAD^+^ ratios to estimate whether the *Δpnp* strain was in a state of higher metabolism than the parent strain W3110 or its complemented strain. Since the persister assay was performed with stationary-phase bacteria, we determined the ATP level in *Δpnp* strain, the W3110 strain, and its complemented strain during the stationary phase. As shown in Fig. 5, the ATP level of *Δpnp* strain was higher than that of the W3110 strain at three time points or that of *Δpnp*-*pnp*+ at 6.5 h and 12 h in stationary phase, suggesting that the higher metabolic status in the *Δpnp* strain produces excessive ATP and renders the *Δpnp* strain less able to form persisters and thus become more susceptible to antibiotics and stresses. Consistent with the above observation, the NADH/NAD^+^ ratio which reflects the redox status of the microbial cells in the *Δpnp* strain was higher than that in the W3110 or its complemented strain at 12 h and 18 h in stationary phase, suggesting that the *Δpnp* strain was indeed in a high metabolic state (Fig. 5).

### Antibiotic exposure assays of metabolism-related gene knockout strains.

**FIG 6:**
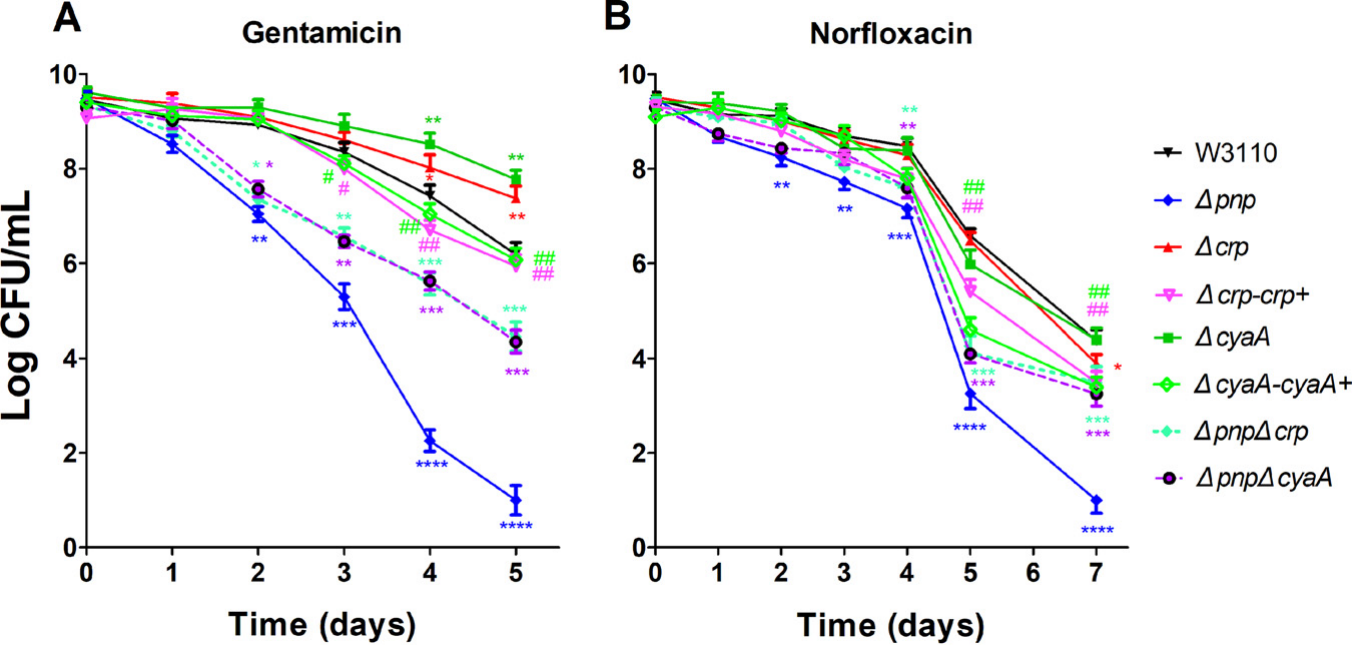
Double-knockout mutants, *ΔpnpΔcrp* and *ΔpnpΔcyaA*, show severe defects in persistence in antibiotic exposure assays. Stationary-phase cultures of the wild-type W3110, *Δpnp*, *Δcrp*, *ΔcyaA*, *Δcrp*-*crp*+, *ΔcyaA*-*cyaA*+, *ΔpnpΔcrp*, and *ΔpnpΔcyaA* strains were exposed to gentamicin (40 μg/mL) (A) and norfloxacin (8 μg/mL) (B), for various times. Aliquots of cultures were taken at different time points, washed, and plated for CFU determination on LB plates. The vertical axis represents CFU values on a log scale, and the horizontal axis represents time of antibiotic exposure in days. Data are the average results from at least three independent experiments. Error bars represent standard deviation (SD). * (strains versus the wild-type W3110) or ^#^ (*Δcrp* versus *Δcrp*-*crp*+ and *ΔcyaA* versus *ΔcyaA*-*cyaA*+), *P < *0.05; ** or ^##^, *P < *0.01; ***, *P < *0.001; ****, *P < *0.0001 (Student’s *t* tests).

The RNA-seq analysis indicated that the impact on bacterial metabolism after *pnp* gene deletion is extensive, and this result suggests that the maintenance of the normal metabolism of bacteria by PNPase may be mediated via global regulators. To address this possibility, we performed qPCR analysis of several major regulators (*arcA*, *arcB*, *cra*, crp, *cyaA*, *fnr*, and *rpoS*) responsible for global regulation of diverse aspects of metabolism in E. coli. Significantly, we found that in the early stationary phase, expression of the *cyaA* encoding adenylate cyclase and *crp* encoding cyclic AMP (cAMP) receptor protein (CRP) increased 3.13- and 3.22-fold, respectively. The other global regulators did not change significantly. Therefore, we knocked out *cyaA* and *crp* and assessed their effect on persister levels in drug exposure experiments. In the killing assays (Fig. 6), persister levels of the *Δcrp* strain and *ΔcyaA* strain were significantly higher than those of the W3110 strain under gentamicin treatment. The persister levels of the *ΔpnpΔcrp* and the *ΔpnpΔcyaA* double-deletion strain under gentamicin and norfloxacin treatment were much higher than that of the *Δpnp* strain. However, complementation experiments with *Δcrp*-*crp*+ and the *ΔcyaA*-*cyaA*+ showed that the persistence of these complemented strains went back to the level of the parent strain W3010 and was significantly different from the *Δcrp* or the *ΔcyaA* strain at three or two time points, respectively.

### Effect of energy inhibitor CCCP on persister levels.

**FIG 7:**
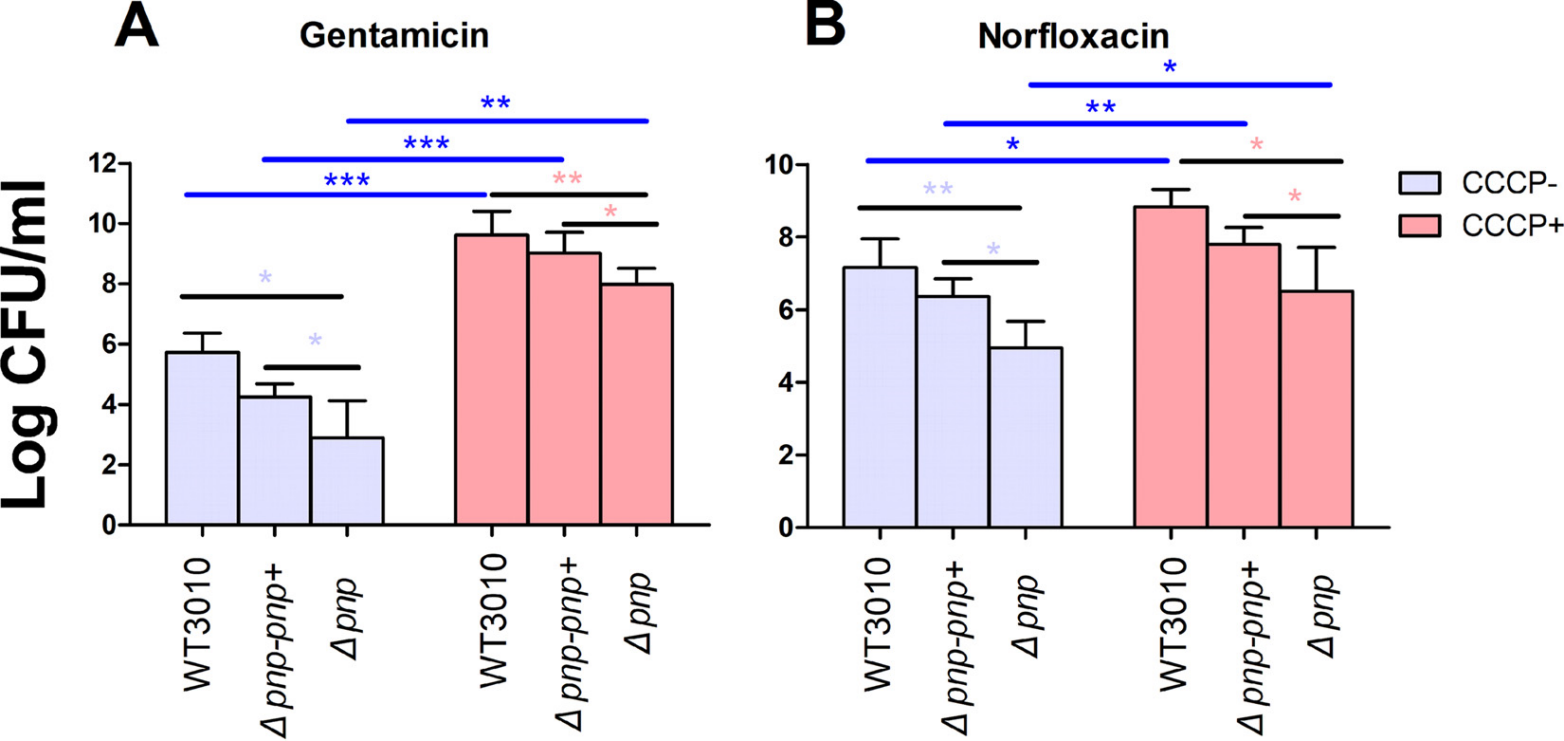
CCCP increases the tolerance of the W3110 strain, the *Δpnp* strain, and its complemented strain (*Δpnp*-*pnp*+) to antibiotics. The W3110 strain, the *Δpnp* strain, and its complemented strain were exposed to gentamicin (40 μg/mL) (A) or norfloxacin (8 μg/mL) (B) with or without CCCP (100 μM) for 8 h. CCCP addition significantly increased the persister level. Gen, gentamicin; Norf, norfloxacin; CCCP, carbonyl cyanide m-chlorophenylhydrazone. Data are the average results from at least three independent experiments. Error bars represent standard deviation (SD). *, *P < *0.05; **, *P* < 0.01; ***, *P < *0.001 (Student’s *t* tests).

In order to further verify that the increased sensitivity to antibiotics was caused by a high level of metabolism in the bacteria, we performed ATP synthesis inhibition experiments using carbonyl cyanide 3-chlorophenylhydrazone (CCCP). CCCP is an inhibitor of oxidative phosphorylation. It can eliminate the transmembrane proton concentration difference in the mitochondrial inner membrane or on both sides of the cell membrane and slow down the oxidative phosphorylation process driven by the proton concentration difference, and one of the effects of CCCP is to inhibit the normal production of ATP. The results showed that the addition of CCCP (100 μM) caused significantly higher tolerance to gentamicin or norfloxacin for the W3110, the *Δpnp* strain, and its complemented strain (Fig. 7), suggesting that the higher persister levels of the three strains were related to the lower metabolism.

### β-Galactosidase activity assay of the *crp*-*lacZ* reporter construct in the W3110, the *Δpnp*, and the *Δpnp*-*pnp*+ strains.

**FIG 8:**
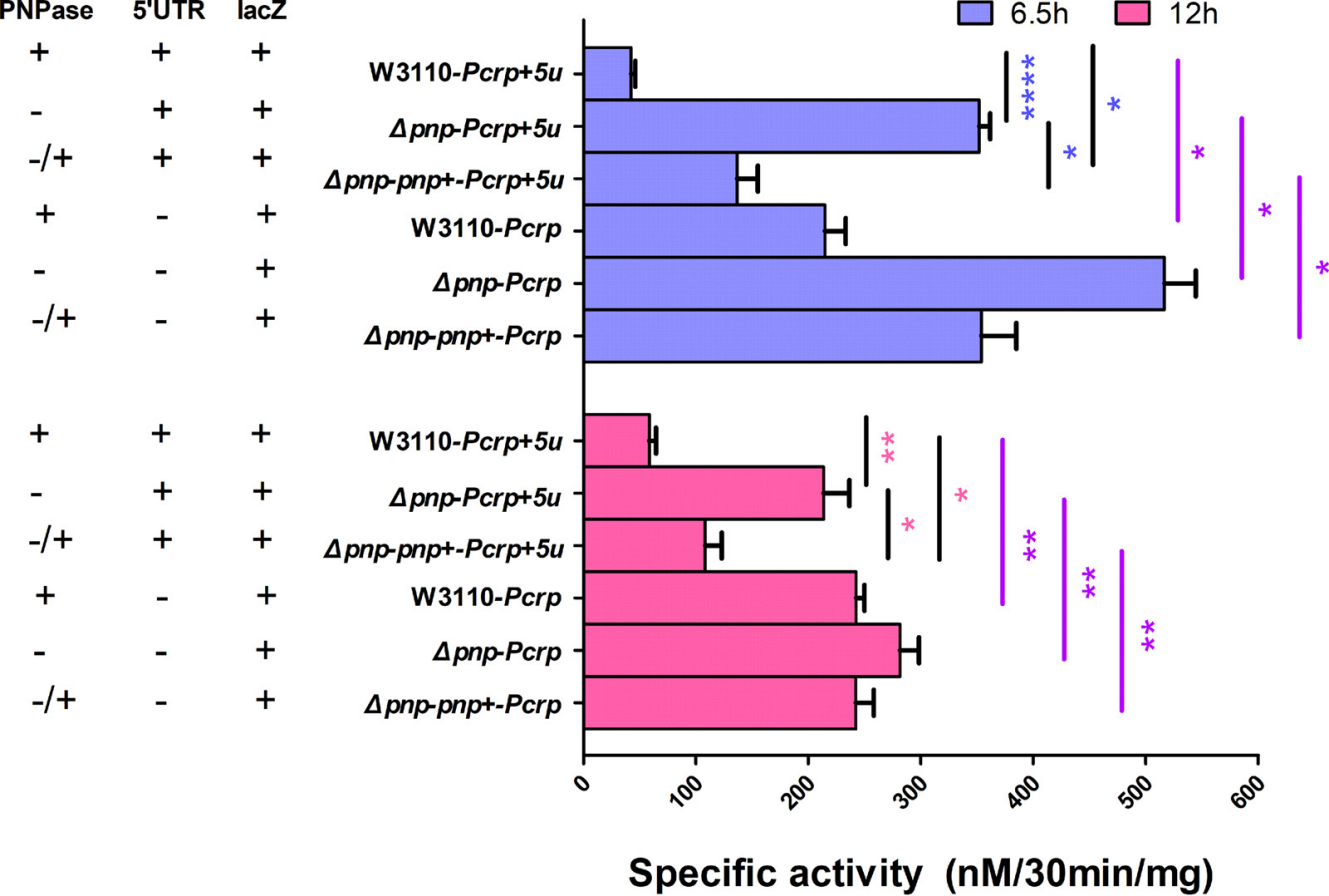
PNPase regulates the transcriptional activity of the *crp* gene through binding to the 5′-UTR of *crp* mRNA. The *crp* transcriptional activity (β-Gal specific activity) in the six strains at two time points of 6.5 h and 12 h was measured. Data are the average results from at least three independent experiments. Error bars represent standard deviation (SD). *, *P* < 0.05; **, *P < *0.01; ***, *P < *0.001; ****, *P < *0.0001 (Student’s *t* tests).

The 5′-UTR region of prokaryotes is one of the important components of posttranscriptional regulation and can affect the initiation of mRNA translation. It has been found that PNPase in E. coli can bind to 5′-UTR region of the operon *pga*ABCD and inhibit the formation of biofilm by inhibiting the expression of acetylglucosamine (22). Therefore, we hypothesized that PNPase may also bind to the 5′-UTR region of *crp* mRNA to inhibit CRP protein translation. To address this hypothesis, we made the *crp-lacZ* reporter construct with or without 5′-UTR (Table 2 and 3) and transformed it into the *Δpnp* strain, the *Δpnp*-*pnp*+ strain, and the parent strain W3110. As shown in Fig. 8, the results showed that the β-galactosidase activity was 8.3-fold higher in *Δpnp-Pcrp + 5u* than that in the parent strain W3110*-Pcrp + 5u* at early stationary phase and 3.6-fold higher at the end of stationary phase. The activity of β-galactosidase in W3110-*Pcrp* was 5.1-fold higher than that in W3110-*Pcrp *+ 5u at early stationary phase, and 4.1-fold higher at the end of stationary phase. The *Δpnp-Pcrp + 5u* strain demonstrated higher β-galactosidase activity than the *Δpnp*-*pnp+-Pcrp + 5u* strain at the two time points (2.6-fold and 1.9-fold, respectively). The activity of β-galactosidase in *Δpnp*-*pnp*+-*Pcrp* was 2.6-fold higher than that in *Δpnp-pnp+-Pcrp + 5u* at early stationary phase and 2.2-fold higher at the end of stationary phase. At any stage, the activity of β-galactosidase in the *Δpnp-Pcrp* was the highest. These findings suggest that PNPase may modulate the translation of CRP mRNA transcripts, leading to a higher metabolic status of the *Δpnp* strain.

**TABLE 2:** Strains and KO primers used in this study

Strain	Gene	KO primer (5′–3′)
*W3110*	*Wild type*	
*Δpnp*	*pnp* KO S	CTGCCCGGTTAAAAGCCCCCCGCCGCAGCGGAGGGCAAATGGCAACCTTAATGGGAATTAGCCATGGTCC
	*pnp* KO A	AGAGGCTTTACCCACATAGAGCTGGGTTAGGGTTGTCATTAGTCGCGAGGGTGTAGGCTGGAGCTGCTTC
*Δcrp*, *ΔpnpΔcrp*	*crp* KO S	AGCGGCGTTATCTGGCTCTGGAGAAAGCTTATAACAGAGGATAACCGCGCATGGGAATTAGCCATGGTCC
	*crp* KO A	GGGAAACAAAATGGCGCGCTACCAGGTAACGCGCCACTCCGACGGGATTAGTGTAGGCTGGAGCTGCTTC
*ΔcyaA*, *ΔpnpΔcyaA*	*cyaA* KO S	GATGTTGGCGGAATCACAGTCATGACGGGTAGCAAATCAGGCGATACGTCATGGGAATTAGCCATGGTCC
	*cyaA* KO A	TCCGCTAAGATTGCATGCCGGATAAGCCTCGCTTTCCGGCACGTTCATCAGTGTAGGCTGGAGCTGCTTC

**TABLE 3:** Plasmids used in this study

Plasmid	Description
pKD3	KO plasmid
pKD46	KO plasmid
pCP20	KO plasmid
pBAD202	Complementary plasmid for *pnp*, *crp*, and *cyaA*
pET28a	pET-lacZ-*Pcrp*/pET-lacZ-*Pcrp + 5u*

## DISCUSSION

In this study, we identified a mechanism of persistence mediated by PNPase, a part of the RNA degradosome. PNPase catalyzes the polymerization of nucleoside diphosphate and the phosphorylation of polynucleotides *in vitro*. But *in vivo*, PNPase is not only the main component of RNA degradosome, which catalyzes the decomposition of RNA and promotes the metabolism and stability of mRNA (24), but also involved in the regulation of bacterial pathogenicity, where PNPase acts as a global regulator of virulence gene expression in Salmonella enterica (25). However, the role of PNPase in the context of persister formation has not been studied. We found that, compared with the wild-type strain, the *Δpnp* strain was highly susceptible to three different antibiotics and stresses, indicating that *pnp* plays an important role in the formation or maintenance of persisters. In order to confirm that PNPase was the direct cause of persister formation, we constructed the *pnp* complementation strain and found that the persistence phenotype was restored in the *pnp* complementation strain. These *in vitro* experiments demonstrate that PNPase participates in persister formation. It is worth noting that the persister-defective phenotypes of the *Δpnp* strain appeared mainly in early to mid-stationary phase. When the cultures were incubated to late stationary phase, the persister-defective phenotypes became less obvious, which indicates that the role of PNPase in persistence likely occurs in persister formation rather than persister survival. In addition, we found that the *Δpnp* deletion strain demonstrated a defect in the motility assay and but more biofilm production than the parent strain W3110, which is consistent with a previous report (22).

To shed new insight on the mechanism by which PNPase mediates persistence, we performed RNA-seq analysis of the *Δpnp* strain compared with that of the W3110 strain, and we found that a number of genes belonging to energy metabolism or virulence pathways are upregulated in the *Δpnp* strain (Fig. 3 and Fig. 4). These findings suggest that elevated metabolism in the *Δpnp* strain is present, which contributes to defect in persistence, as shown by increased susceptibility to antibiotics and stresses. This idea is confirmed by the measurement of the levels of ATP and NADH/NAD^+^, which are two indicators of redox state in bacteria, indicating that the *Δpnp* strain had significantly higher metabolism than that of the W3110 parent strain and was highly susceptible to antibiotics and stresses even in stationary phase. On the other hand, our RNA-seq data also showed 15 genes enriched in the ribosome pathway were all downregulated, suggesting that in the *Δpnp* strain protein synthesis is suppressed to a certain extent. PNPase is a major player in bacterial RNA turnover and, meanwhile, also has a protective role in mRNA stability (24, 26). It is likely that the RNA degradation defect or/and regulatory function independent of the RNA degradosome of PNPase regulates the protein synthesis via an unclear feedback loop, which needs further study.

The E. coli CRP is an important transcription factor which can result in positive or negative regulation of more than 100 genes involved mainly in the catabolism of carbon sources other than glucose (27–29). In our study, the results of the validation of differentially expressed genes showed that the mRNA levels of genes encoding, for example, carbohydrate transport (*mglBAC*, *lamB*, and *malK*), TCA cycle (*sdhA*, *sdhD*, *fumA*, *mdh*, and *acnB*), and glycerolipid metabolism (*glpD*, *glpK*, and *glpT*) were significantly upregulated. Interestingly, the metabolic master regulator CRP has a positive regulatory effect on these genes as shown in previous studies (1, 29). In addition, the cAMP-CRP complex is known to be involved in the regulation of biofilm formation, quorum sensing systems, and transcription of the nitrogen regulatory system (30–32). When the culture environment lacks glucose, CRP binds to the effector cAMP, and the activated CRP in turn binds to the TGTGAnnnnnnTCACA sequence near the promoter of the regulatory gene, thereby recruiting RNA polymerase to initiate the transcription of the downstream genes (33). CRP was found to play an important role in the metabolic process in E. coli, and the perturbation of cAMP-CRP can significantly diminish the metabolic capabilities of persisters (34). We knocked out the *crp* and *cyaA* gene in the W3110 and the *Δpnp* strain and found that the ability of persister formation was significantly higher in the double-deletion strains *ΔcrpΔpnp* and *ΔcyaAΔpnp* than that in the single-deletion strain *Δpnp*. This finding is consistent with the recent study that the *Δcrp* mutant conferred tolerance to killing by a variety of antibiotics (35). PNPase can bind to its own 5′-UTR region to realize the autologous expression regulation by promoting degradation of *pnp* mRNA in the presence of RNase III (36–38). We inferred that PNPase in E. coli can inhibit the translation of the *crp* mRNA, which controls the CRP level in the normal range, to maintain the balance of intracellular metabolism. To confirm this inference, we measured the activity of β-galactosidase to reflect the transcriptional activity of *crp* in the W3110 strain and the *Δpnp* strain. We found that the transcriptional activity of *crp* in the *Δpnp* strain was indeed increased 8.3-fold in the early stationary phase, indicating that PNPase can regulate the translation of the CRP protein at the posttranscriptional level and that PNPase deficiency leads to elevated metabolism and reduced persistence.

**FIG 9:**
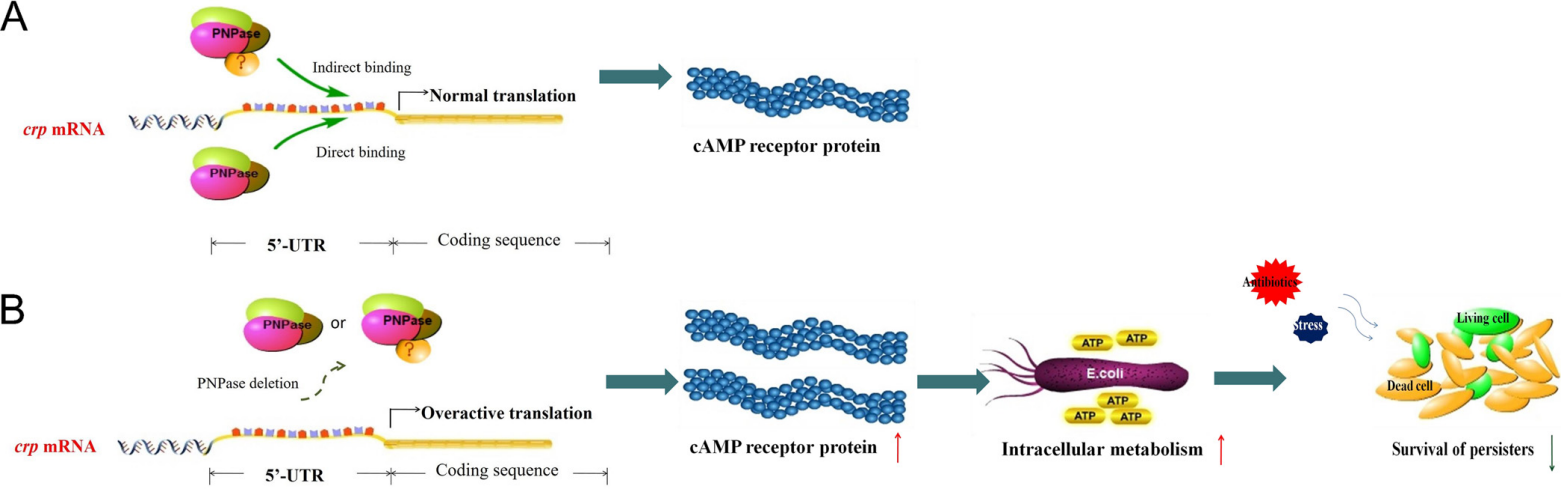
PNPase regulates the formation of bacterial persisters through CRP directly or indirectly. (A) PNPase binds to the 5′-UTR of *crp* mRNA to cause a negative regulation of CRP protein, which facilitates the downregulation of metabolism to allow persister formation. (B) PNPase deletion leads to overexpression of CRP and higher metabolism as seen by elevated redox (NADH/NAD^+^ ratio) and ATP levels, resulting in a failure to enter the dormant state and thus causing decreased persister formation.

The metabolic status is critical in persister formation in bacteria (39–42). Based on our findings, we propose that PNPase in E. coli can bind to the 5′-UTR of the *crp* mRNA transcript, directly or indirectly, to cause a negative regulation of CRP protein, which facilitates the downregulation of metabolism to allow persister formation, and that the deletion of PNPase could cause the overexpression of *crp* and hypermetabolism, leading to a failure to enter the dormant state and causing decreased persistence, as shown by its inability to tolerate antibiotics and stress conditions in the *Δpnp* deletion strain even in stationary phase (Fig. 9). This result is consistent with the previous observation that the inability to enter low metabolic state in the PhoU mutant also leads to hypermetabolism with a defect in persister formation and a higher susceptibility to antibiotics and stresses in the stationary phase (43).

In summary, our study established a mechanism of persister formation mediated by PNPase regulation of metabolic activity in E. coli (Fig. 9). We found that PNPase controls cellular metabolism by negatively regulating the global regulator cyclic AMP receptor protein (CRP) operon at the posttranscriptional level via targeting the 5′-UTR region of the *crp* transcript to regulate persister formation. The results of the *in vitro* studies need to be further tested in animal models to determine if PNPase has any role in persistence and virulence in the future. The findings that PNPase is a regulator of persistence in this study and a target of tuberculosis persister drug PZA (12, 13) suggest that PNPase in different bacteria could be a promising new drug target for developing novel persister drugs for the improved treatment of persistent bacterial infections.

## MATERIALS AND METHODS

### Bacterial strains and growth media.

The strains used in this work were derived from wild-type E. coli K-12 W3110 [F−mcrAmcrBIN(rrnD-rrnE)1 lambda−]. Luria-Bertani (LB) broth (0.5% NaCl) and agar were used for bacterial cultivation.

### Construction of E. coli W3110 knockout mutants.

The λ red recombination system was used for construction of the *pnp* knockout mutation in the E. coli chromosome as described (44). The candidate gene was replaced by the chloramphenicol resistance gene and cloned into plasmid pCP20 for constructing the knockout mutant. Primers used for knockout and additional external primers used to verify the correct integration of the PCR fragments by homologous recombination are shown in Table 2.

### Construction of pBAD202 recombinant plasmids.

The plasmid pBAD202 was used for construction of a recombinant containing a functional wild-type gene for complementation (45). The primers designed based on the *pnp* gene were *pnp*-F (5′-CATGCCATGGCAATGCTTAATCCGATCGTTCGTAA-3′) and *pnp*-R (5′-CCCAAGCTTGCAAATGGCAACCTTACT-3′). Similarly, the *crp* and *cyaA* recombinant plasmids were constructed with the primers *crp*-F (5′-GCTCTAGAGCATGGTGCTTGGCAAACCGCAAACA-3′) and *crp*-R (5′-CGGAATTCGCGCCACTCCGACGGGATTAACGA-3′) and the primers *cyaA*-F (5′-GCTCTAGAGCATGTACCTCTATATTGAGACTCTGA-3′) and *cyaA*-R (5′-CGGAATTCCGTCACGAAAAATATTGCTGTAATAG-3′), respectively. The PCR products were digested and ligated to the plasmid pBAD202. The recombinant construct containing the *pnp* gene was used to transform the *Δpnp* strain, the *Δpnp*-*pnp*+ strain, and the wild-type W3110 strain by electroporation for complementation of these mutants. The recombinant construct containing the *crp* or *cyaA* gene was used for a rescue experiment for the *Δcrp* strain or the *ΔcyaA* strain.

### Persister assay for antibiotics.

Persisters are rare phenotypic variants within a bacterial population that are capable of tolerating lethal antibiotic concentrations. Therefore, the concentrations of antibiotics used in the persister assays are usually more than 10 times the MIC from the literature and also from our own pilot experiments. Persistence was measured by determining bacterial survival in the form of CFUs upon exposure to three antibiotics, namely, ampicillin at 200 μg/mL, norfloxacin at 8 μg/mL, and gentamicin at 40 μg/mL. E. coli cells were grown to stationary phase in LB medium and then were exposed to different antibiotics, where undiluted cultures were used for incubation without shaking at 37°C for various times. The number of CFUs per milliliter was determined by plating dilutions of the bacterial cells on LB plates without antibiotics. The different stresses or antibiotics had different killing effects, so the time points were also different.

### Persister assay for stresses.

The antibiotic concentrations, temperature, osmotic pressure, pH, and concentrations of hydrogen peroxide used in this study are based on the results of our pilot experiments. For heat stress, E. coli cells from stationary-phase cultures were treated at 52°C for 30 min without shaking. The CFUs were determined after serial dilutions. For acid stress, E. coli cells were incubated with acid of pH 3 for 4 days at 37°C without shaking. For hypertonic saline stress, cultures were grown in LB medium containing 3 M NaCl at 37°C for 6 days without shaking. E. coli cells were also exposed to 80 mM H_2_O_2_ at 37°C for 5 days without shaking. The number of CFUs under acid, hypertonic saline, and H_2_O_2_ conditions was determined daily. Shaking cultures were used from logarithmic phase to stationary phase, while nonshaking cultures for drug exposure persister assays, which are used commonly in the field (46).

### RNA extraction and RNA sequencing.

The *Δpnp* strain and the wild-type W3110 strain were cultivated at 37°C for 6.5 h to stationary phase. Total RNA was isolated from a 1-mL culture, using the RNeasy minikit (Qiagen, USA), according to the manufacturer’s instructions. RNAs derived from three independent experiments were used for RNA-seq. All procedures for RNA sequencing and alignment of the transcriptome were conducted by OEbiotech (Shanghai, China). An Agilent 2100 bioanalyzer was used to qualify the sample library. RNA sequencing was performed using the Illumina HiSeq 2000 platform. Raw reads were filtered to remove low-quality sequences, adapter sequences, and reads with poly N. The clean reads were mapped to the reference genome using the TopHat package and to reference genes using Bowtie 2 software (47, 48). Differential expression analysis of two samples was performed using the software DEseq (49). The David Bioinformatics Resource 6.7 (https://david.ncifcrf.gov/) online database was used to perform gene ontology and KEGG pathway analysis.

### Detection of bacterial internal redox status.

Based on the growth curve (Fig. S1A), the time points 6.5 h, 12 h, and 18 h were defined as the early stationary phase, the middle stationary phase, and the end of stationary phase, respectively. Therefore, ATP and NAD+/NADH assays were performed at three time points. The concentration of intracellular ATP was detected by a BacTiter-Glo microbial cell viability assay kit (Promega, USA) according to the manufacturer’s instructions (50). The intracellular NADH/NAD^+^ ratio was measured as described (51). Carbonyl cyanide m-chlorophenylhydrazone (CCCP), an oxidative phosphorylation inhibitor, was purchased from Sigma-Aldrich (St. Louis, MO). CCCP (100 μM) and antibiotics were added to stationary-phase cultures (52), and the number of CFUs per milliliter was determined by plating serial dilutions of bacteria onto LB plates without antibiotics after 24 h of incubation at 37°C with shaking at 210 rpm.

### Detection of transcriptional activity of cyclic AMP (cAMP) receptor protein (CRP).

The β-galactosidase gene *lacZ* was inserted into the multiple cloning site of the pET-28 vector, and the original T7 promoter was replaced with the promoter region of the *crp* gene or promoter region of the *crp* gene plus 5′-UTR region. The recombinant plasmid constructs pET-*lacZ*-*Pcrp*/pET-*lacZ*-*Pcrp *+* 5u* were transformed into the W3110 strain, the *Δpnp* strain, and the *Δpnp*-*pnp*+ strain by electroporation. The activity of β-galactosidase was measured by the β-Gal assay kit (Invitrogen, USA) according to the manufacturer’s instructions.

### Statistical analysis.

At least three independent biological replicates were used for all experiments. Each data point in the figures represents the mean value ± standard deviation (SD). Statistical analyses were performed using GraphPad Prism 8.4.3 software. The significance of experimental differences in the persister assay, ATP assay, redox status assay, and β-Gal assay was evaluated with the two-tailed Student’s *t* test. The *P* value threshold was selected as follows: * or ^#^, *P < *0.05; ** or ^##^, *P < *0.01; ***, *P < *0.001; and ****, *P < *0.0001.

### Data availability.

All the raw data from RNA-seq were submitted to the NCBI Sequence Read Archive and deposited under the accession number PRJNA846098.
