# Supplementary material for: Polynucleotide Phosphorylase Mediates a New Mechanism of Persister Formation in Escherichia coli
Source: Microbiol Spectr. 2022 Dec 8;11(1):e01546-22. doi: 10.1128/spectrum.01546-22 (PMC9927094; doi:10.1128/spectrum.01546-22)
Supplement: Supplemental file 3 — Fig. S1 and S2. Download spectrum.01546-22-s0003.pdf, PDF file, 0.8 MB [file spectrum.01546-22-s0003.pdf]

Polynucleotide Phosphorylase (PNPase) Mediates a New Mechanism of Persister Formation in *Escherichia coli*

Nan Wu[a,b], Yumeng Zhang[b], Shanshan Zhang[b], Youhua Yuan[b], Shuang Liu[b], Tao Xu[b], Peng Cui[b], Wenhong Zhang[b*] and Ying Zhang[b,c*]

[a] Department of clinical laboratory, Shanghai Stomatological Hospital, Shanghai, China

[b] Department of Infectious Diseases, Shanghai Key Laboratory of Infectious Diseases and Biosafety Emergency Response, National Medical Center for Infectious Diseases, Huashan Hospital, Fudan University, Shanghai, China

[c] State Key Laboratory for the Diagnosis and Treatment of Infectious Diseases, The First Affiliated Hospital, Zhejiang University School of Medicine, Hangzhou, China

* Correspondence:

Ying Zhang

yzhang207@zju.edu.cn

Wenhong Zhang

zhangwenhong@fudan.edu.cn

Supplemental Figures

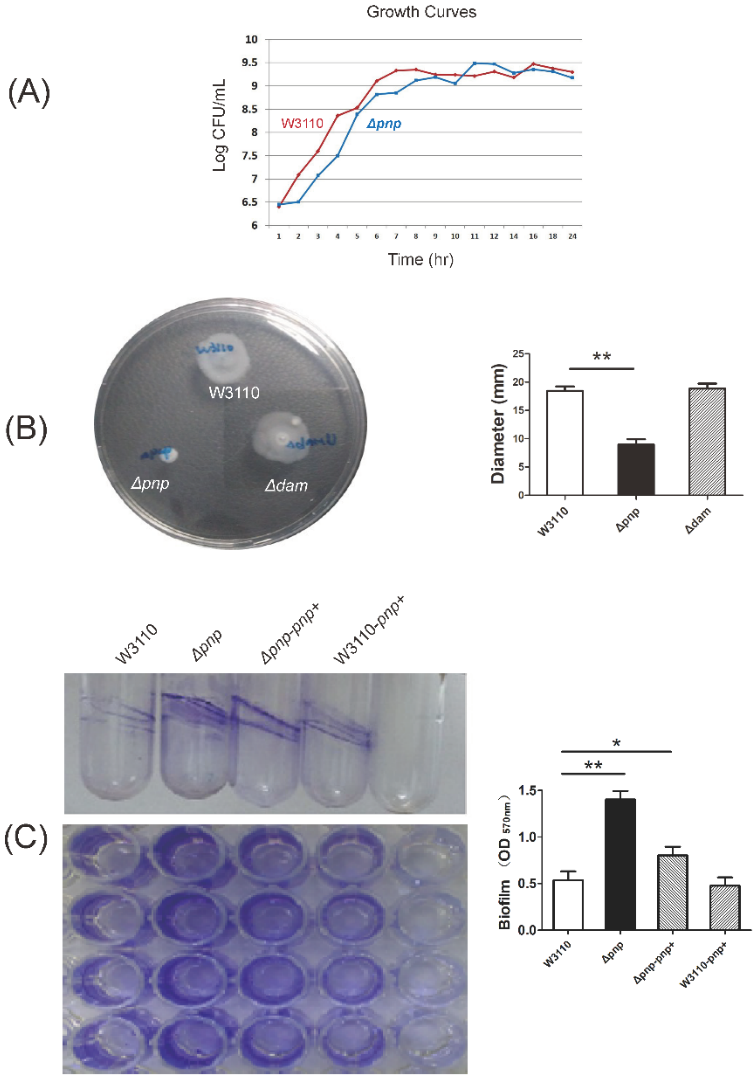

FIG S1 Growth curve, motility assay, biofilm formation of the *pnp* mutants and the W3110 strain. (A) Growth curve for the *Δpnp* and the W3110 (n = 3 for each strains). (B) Motility assay for the *Δpnp*, W3110 and *Δdam* (a mutant involved in persistence but normal in motility assay) strains performed in LB containing 0.1% agar (n = 4 for different strains) and their respective quantitation in bar chart on the right hand side. (C) Biofilm formation assay carried out in a 15 mL glass tube (upper panel) and a flat bottomed 96-well plate (bottom panel) (n = 4 for different strains grown in LB) and their respective quantitation in bar chart on the right hand side. Data are the average results from at least three independent experiments. Error bars represent standard deviation (SD). *, $P < 0.05$; **, $P < 0.01$ (Student's *t* tests).

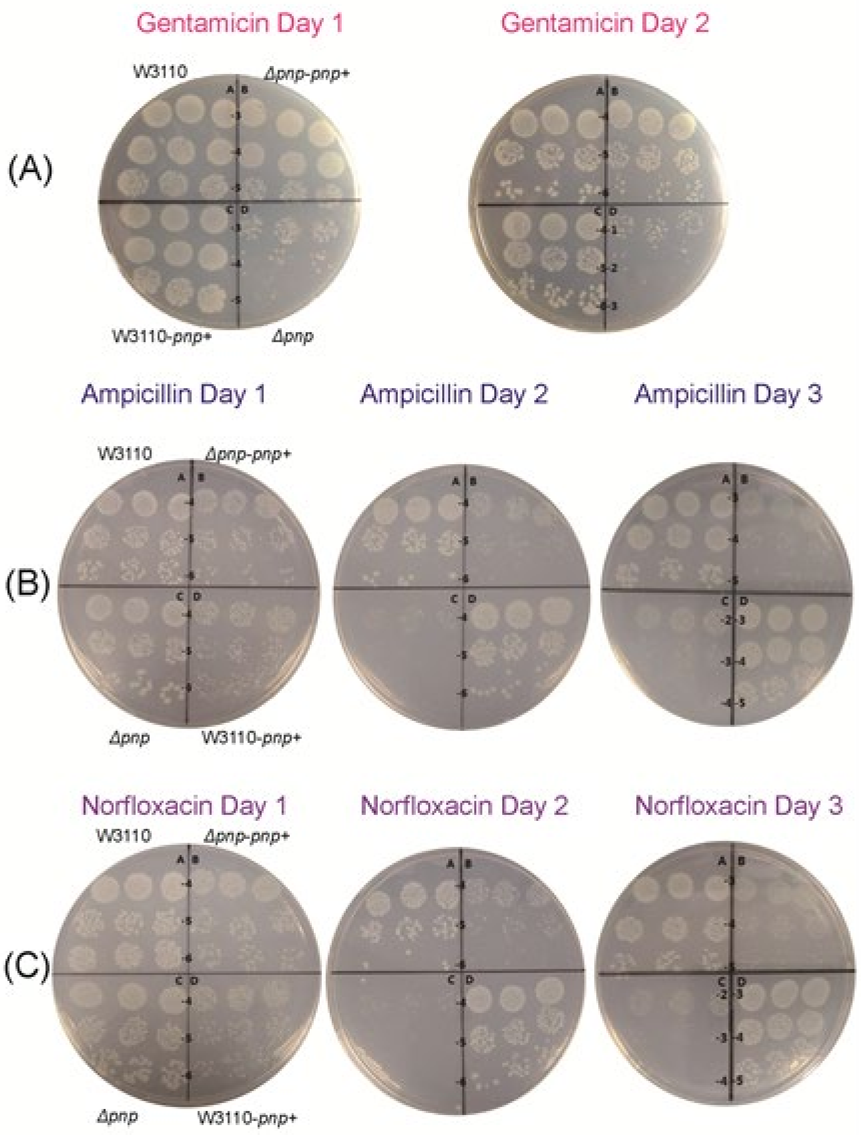

FIG S2 Phenotypes of the *pnp* mutants and the W3110 strain related to Fig 1. (A) Early stationary phase cultures of the *pnp* deletion strain (*Δpnp*), its complemented strain (*Δpnp-pnp*+), over-expression strain (W3110-*pnp*+) and the W3110 were exposed to gentamicin (40 μg/mL) for 1 day and 2 days. (B) Early stationary phase cultures of the *pnp* deletion strain, its complemented strain, over-expression strain and the W3110 were exposed to ampicillin (200 μg/mL) for 1 day, 2 days and 3 days. (C) Early stationary phase cultures of the *pnp* deletion strain, its complemented strain, over-expression strain and the parent strain W3110 were exposed to norfloxacin (8 μg/mL) for 1 day, 2 days and 3 days. Numbers on the dish：dilution ratio.
